# Optical Bistability in Photonic Topological Hypercrystals and Its Applications in Photonic Neural Network

**DOI:** 10.3390/nano16090561

**Published:** 2026-05-02

**Authors:** Hanli Li, Boyang Duan, Tianyu Zhu, Sichao Shan, Liqian Lin, Changjun Li, Zhitong Li

**Affiliations:** 1 School of Computer Science, Beijing University of Posts and Telecommunications, Beijing 100876, China; 2 State Key Laboratory of Information Photonic and Optical Communication, Beijing University of Posts and Telecommunications, Beijing 100876, China; 3 Queen Mary School Hainan, Beijing University of Posts and Telecommunications, Beijing 100876, China; 4 School of Information and Communication Engineering, Beijing University of Posts and Telecommunications, Beijing 100876, China; 5 School of Physical Science and Technology, Beijing University of Posts and Telecommunications, Beijing 100876, China

**Keywords:** hyperbolic metamaterials, photonic neural network, optical bistability

## Abstract

Optical bistability is a nonlinear phenomenon enabling stable switching between two optical states and has important applications in optical communication and photonic neural networks (PNNs). However, conventional bistable devices often suffer from fabrication imperfections and scattering losses, which limit their robustness and dispersionless performance. In this study, we numerically investigate optical bistability from a one-dimensional photonic topological hypercrystal (PhH) composed of alternating hyperbolic metamaterials (HMMs) and dielectric layers. By designing a center-inversed symmetric layered PhH structure and introducing Kerr nonlinearity into the localized dielectric region of maximum electric field intensity at the inversion center, we achieve a robust, angle-insensitive optical bistability for TM polarization through phase variation compensation mechanism. When applied as a nonlinear activation function in PNNs, the bistable PhH exhibits performance comparable to conventional digital activation functions such as ReLU and Sigmoid in image-recognition tasks. Our work paves the way for integrating topological bistable devices into next-generation PNNs.

## 1. Introduction

Optical bistability is a nonlinear phenomenon in which an optical system can stably switch between two stable output states under the same input, giving rise to an optical hysteresis loop. In Kerr-type bistability, this behavior originates from an intensity-dependent permittivity, so achieving a pronounced bistable response typically requires both a large Kerr nonlinearity and strong field enhancement. Therefore, it exhibits great potentials for making optical switches, storage devices, and modulators, which are increasingly important for optical communication and information processing [1,2,3]. Recently, its nonlinear property has also been investigated as a nonlinear activation function in developing the photonic neural networks (PNNs) [3,4,5,6,7]. However, bistable operation relies on a well-defined and stable optical mode: fabrication tolerances and surface roughness can shift resonance wavelengths, while scattering and coupling perturbations can distort the effective input–output conditions [8]. Therefore, developing highly robust and dispersionless optical bistable devices and neural network is always an ongoing pursuit. Recent advances in optical artificial intelligence, intelligent nanophotonics, and optical neural networks have further highlighted the importance of compact, physically implementable optical nonlinear elements for future AI hardware [9,10,11].

Photonic hypercrystals (PhHs), formed by alternating hyperbolic metamaterials (HMMs) and dielectric layers, provide a promising route to realize angle-robust band gaps and cavity modes. Because the HMM exhibits hyperbolic dispersion, the phase accumulated in the HMM layer can counterbalance that in the dielectric layer as the incidence angle varies. This phase variation compensation stabilizes the unit-cell Bloch phase, making the band gap and the embedded cavity resonance only weakly dependent on the incidence angle. This is favorable for dispersionless cavity modes and their bistable phenomena [6,7,12,13,14].

In this paper, we investigate the feasibility of achieving a dispersionless nonlinear optical phenomenon within a simple one-dimensional topological PhH. We design a PhH material structure with a non-dispersive property and endow it with a center-inversed symmetry. Compared to the mirror symmetric structure of PhH, a resonant peak appears in the energy band gap. We study the non-dispersive nature of this resonant peak and the energy band gap by varying the incident angle. When we replace several layers of dielectric materials, where the electric field distribution is strongest, with nonlinear materials in this structure, we achieved a robust dispersionless optical bistable system. Additionally, we simulate the bistable characteristics as activation functions in a convolutional neural network, demonstrating that the bistable nonlinear activation function is comparable to the traditional nonlinear activation functions, such as ReLU and Sigmoid, in image recognition accuracy.

## 2. Materials and Methods

**Figure 1:**
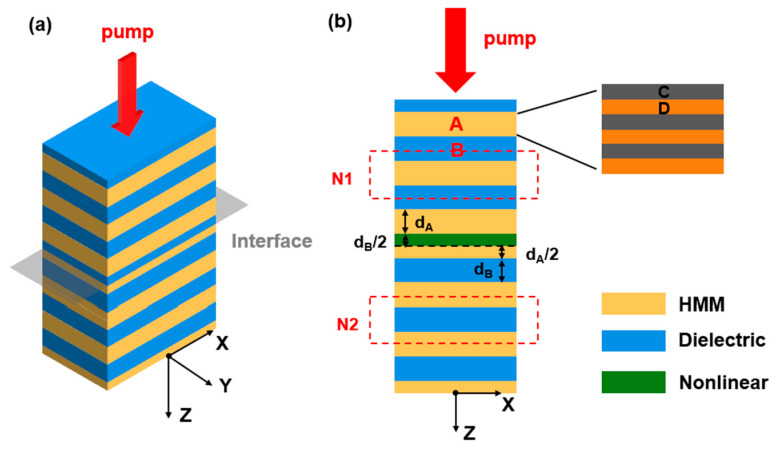
(**a**) A three-dimensional schematic diagram of the proposed PhH consisting of alternating HMM layers and isotropic dielectric layers. (**b**) A longitudinal cross-sectional view of the PhH structure. The nonlinear response is introduced only into the central silicon layer at the inversion center, highlighted in green. The HMM layer A is simulated with a subwavelength unit cell composed of a metal–dielectric layer substructure ${\left(CD\right)}_{S}$. $d_{A}=S(d_{C}+d_{D})$. The background is air.

As shown in Figure 1, the proposed PhH is a center-inversed symmetric layered structure. The symmetric center is located at the mirror interface marked in Figure 1a. This PhH consists of alternating thin film layers of HMM and isotropic materials on both sides of the symmetric center, as depicted in Figure 1. The HMM medium A, represented in yellow in Figure 1b, is described by an anisotropic dielectric constant tensor of ($\varepsilon_{Ax}$, $\varepsilon_{Ay}$, $\varepsilon_{Az}$), and the medium B, represented in blue, has an isotropic dielectric constant of $\varepsilon_{B}$. Both sides from the symmetric center exhibit a periodic layer structure, but the arrangement order of the layers within each period is different. The upper side has a sequence of N1(B-A-B), and the lower side has a sequence of N2(A-B-A). In this section, material loss is included, so the dielectric constants are complex-valued. Let $d_{A}$ and $d_{B}$ represent the thicknesses of the two cladding layers, and $d=d_{A}+d_{B}$ denote the total length of the unit cell. We assume that media A and B are non-magnetic, i.e., $\mu_{A}=\mu_{B}=1$. T denotes the number of periods on one side of the symmetric center, and the surrounding medium is air.

We consider a TM-polarized plane wave incident in the x–z plane at an angle θ, as shown in Figure 1a. The magnetic field vector is oriented in the y-direction. Owing to the planar invariance, the in-plane wave vector component $k_{x}$ is conserved across all layers. In the uniaxial HMM layer A (optical axis along z), the TM field excites only the extraordinary wave, so the out-of-plane component $k_{Az}$ is governed by the anisotropic (hyperbolic) dispersion relation, whereas in the isotropic layer B the corresponding component $k_{Bz}$ follows the usual isotropic dispersion. In these expressions, the wave vector in vacuum is given by $k_{0}=\omega /c$. When $\varepsilon_{Ax}>0$ and $\varepsilon_{Az}<0$, layer A exhibits hyperbolic dispersion with the optical axis along z. In contrast, the TE-polarized wave does not experience the same hyperbolic phase variation compensation in the present structure, so its angular response is much less stable. Therefore, the following analysis focuses on TM-polarization.

The first band gap of the PhHs is determined by Bragg reflection, i.e., the total phase accumulated over one unit cell reaches the first-order Bragg condition at the Bragg frequency $\omega_{Brg}$. As the incident angle increases, $k_{x}$ increases. In the isotropic layer B this reduces $k_{Bz}$, while in the hyperbolic layer A the extraordinary-wave component $k_{Az}$ increases with $k_{x}$. These opposite angular trends allow the phase accumulated in A to compensate that in B, leading to an angle-insensitive band gap around the design frequency, this compensatory effect is referred to as the phase variation compensation effect. In practice, we choose $d_{A}$ and $d_{B}$ so that the first-order angular variation in the unit-cell phase vanishes at the design frequency, yielding a thickness-ratio condition determined by $\varepsilon_{Ax}$, $\varepsilon_{Az}$, and $\varepsilon_{B}$. More quantitatively, if the total propagating phase in one unit cell at the Bragg frequency is written as $\Phi =k_{Az}d_{A}+k_{Bz}d_{B}$, the suppression of angular dispersion requires $\partial \Phi /\partial k_{x}=d_{A}\left(\partial k_{Az}/\partial k_{x}\right)+d_{B}\left(\partial k_{Bz}/\partial k_{x}\right)=0$, which is the phase-compensation condition given by Equation (5) in Ref. [12]. Under TM polarization, the extraordinary wave in the HMM yields $\partial k_{Az}/\partial k_{x}>0$, whereas the isotropic dielectric layer gives $\partial k_{Bz}/\partial k_{x}<0$; therefore, when $d_{A}$ and $d_{B}$ are properly chosen, the opposite phase variations cancel each other to first order. This first-order cancelation provides the analytical basis for the suppressed angular shift in the Bragg frequency, and thus for the nearly angle-insensitive gap and resonant wavelength observed in Figure 3.

According to the effective medium theory, a one-dimensional metal–dielectric layer with subwavelength unit cells can be equivalently described as a hyperbolic metamaterial (HMM). A magnified view of the HMM layer A with substructure ${\left(CD\right)}_{S}$ is shown in Figure 1, where ${\left(CD\right)}_{S}$ represents a subwavelength metallic-dielectric multilayer, with the thickness of layer C being $d_{C}$ and the thickness of layer D being $d_{D}$. Here, S denotes the number of periods of ${\left(CD\right)}_{S}$. Therefore, we express ${\left(AB\right)}_{N}$ as ${{\left[(CD\right)}_{S}B]}_{N}$. As mentioned earlier, dielectric medium B should have a high refractive index. Thus, we choose dielectric materials B and C to be silicon (Si), with $n_{Si}=3.48$ (which means the dielectric constant $\varepsilon_{Si}={n_{Si}}^{2}=12.11$). Tin-doped indium oxide (ITO) is selected as the metallic material as it exhibits plasmonic properties in the infrared and visible light regions. The plasma frequency of ITO can be designed by controlling the doping level of ${Sn}^{4+}$. The dielectric constant of ITO is described using the Drude model.

$$\varepsilon_{D}\left(\omega \right)=\varepsilon_{\infty }-\frac{\omega_{pD}^{2}}{\omega^{2}+j\omega \gamma D},$$

where $\varepsilon_{\infty }$ is the background permittivity, $\omega_{pD}={\left(N_{0}e^{2}/\varepsilon_{0}m^{*}\right)}^{1/2}$ is the plasma frequency, and γD is the damping frequency. In this work, we set $\varepsilon_{\infty }=3.9$, $m^{*}=0.4m_{e}$, and $\omega_{pD}=2.48\;eV$.

Based on the effective medium method, the substructure ${\left(CD\right)}_{S}$ can be homogenized into an anisotropic medium with $\varepsilon_{Ax}=\varepsilon_{Ay}$ given by the thickness-weighted (arithmetic) average of the constituent permittivity, and $\varepsilon_{Az}$ given by the corresponding harmonic average. The filling ratio p denotes the volume fraction of layer D within one C/D period. The filling ratio $p=d_{D}/(d_{C}+d_{D})$ represents the volume percentage of the D layer in the substructure unit cell. We assume $d_{C}=d_{D}=25\;nm$, thus p=0.5. The optical frequency (ω/2π) varies around 200 THz, corresponding to a wavelength of about 1500 nm, which is much larger than the thickness of the unit cell $d_{C}+d_{D}$. Therefore, the effective medium method is valid. In the frequency range of 150 to 300 THz, we have $\varepsilon_{Ax}>0$ and $\varepsilon_{Az}<0$, which corresponds to HMM. Additionally, within the region of 150 to 220 THz, $|\varepsilon_{Az}|\gg 1$, and we will design $\omega_{Brg}$ in this range.

After obtaining the dielectric constants of the materials in the ${\left(AB\right)}_{N}$ structure, we will further determine $d_{A}$ and $d_{B}$. According to the dispersionless condition and considering that HMM layer A is composed of the substructure ${\left(CD\right)}_{S}$ with thicknesses $d_{C}=d_{D}=25\;nm$, we choose S=4, which corresponds to $d_{A}=200\;nm$. Under these conditions, the corresponding frequency is $\omega_{Brg}/2\pi =207.2\;THz$, with $d_{B}=96\;nm$. Of course, the other parameters can be selected as long as the parameters of the substructure ${\left(CD\right)}_{S}$ are predetermined.

At the interface of two PhHs, the existence of localized topological states arises from the mismatch of their surface impedances, which is closely related to the geometric phase (Zak phase) of the PhH on both sides of the interface. The Zak phase is a quantized topological constant that can take values of 0 or π described by the following equation $\exp\left(i\theta_{Zak}\right)=sign\left[1-\frac{\mu_{s}\varepsilon_{c}}{\mu_{c}\varepsilon_{s}}\right]=sign[\eta ]$, where η represent the impedance and μ and ε represent the permittivity and permeability of the material [15]. When the PhHs on either side of the interface have opposite-sign surface impedances, topological states can form. In our designed 1D photonic hypercrystal, although the PhHs on both sides of the inversion-symmetric center have the same band structure, they possess different Zak phases, enabling the formation of topological states at the interfaces of the two PhHs [16].

**Figure 2:**
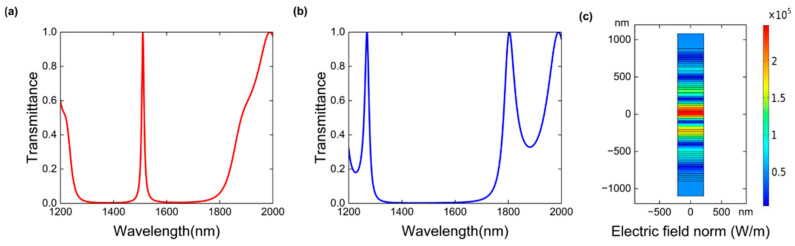
(**a**) The transmission spectrum of the center inversion-symmetric structure. (**b**) The transmission spectrum of the mirrored symmetric layered structure. (**c**) Electric field distribution of the center-inversed symmetric structure at the peak wavelength.

Based on the aforementioned parameters, we calculate the band structure. Figure 2a,b show the transmission spectra of the proposed center-inversed symmetric layered structure and the periodic layered structure with a period T=3 at low incident light intensity ($P_{in}=1\;W/m$), respectively. The band gaps of both of the structures are the same, spanning from 1300 to 1800 nm. But in the center-inversed symmetric structure, a resonant peak appears at 1515 nm with full width at half maximum (FWHM) of 6.8 nm, indicative of topological edge mode. When the period T increases, the wavelength of that peak remains, while the full-width at half-maximum (FWHM) decreases. To better observe the bistable states discussed in the following, the period T=3 is used in all the PhH simulation. As depicted in Figure 2c, the electric field intensity of the transmission peak is highly localized at the inversion center of the layered structure, and decays exponentially into the bulk of the layers on both sides, exhibiting a typical topological boundary state distribution [17]. The topological properties of the resonance peak remain intact when the period is changed, with the electric field confined between the two optical bands, resulting in the wavelength of the topological resonance mode not varying with the period, and the electric field intensity reaching its peak and decaying exponentially at this location.

**Figure 3:**
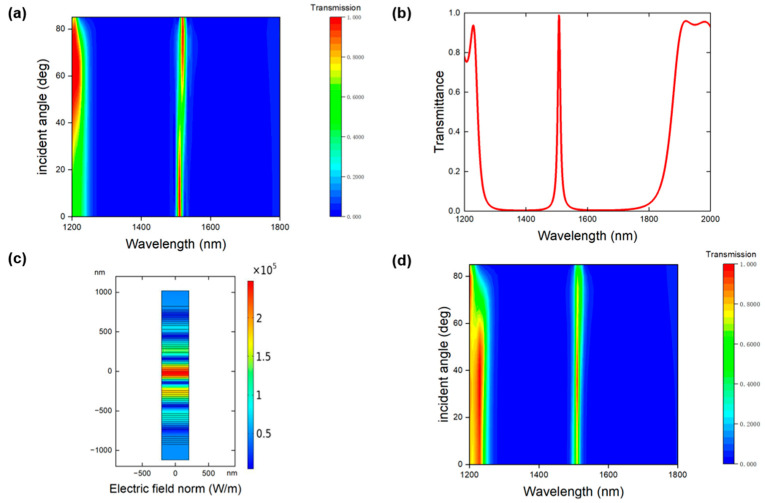
(**a**) Simulated transmission spectrum of PhH as a function of incident angle for TM polarizations. (**b**) Transmission spectrum when the perturbation is induced in the thickness of each layer. (**c**) Electric field distribution at the peak wavelength in (**b**). (**d**) Transmission spectrum versus incident angle for the structure with random thickness variations under TM polarization.

As shown in Figure 3, we obtain the transmission spectrum of the PhH structure by varying the angle of incidence of the input light under TM polarization. Due to the phase variation compensation effect, it can be observed that under TM conditions, a stable band gap appears between 1250 nm and 1800 nm. Within this band gap, the transmission peak remains at the wavelength around 1510 nm as the angle of incidence increases. This occurs because, under TM conditions, the electric field component E along the y-axis increases with the angle of incidence. To test the robustness of the topological optical state, we introduce perturbations into the thickness of each layer. Specifically, each layer is randomly changed by a 10% in thickness, mimicking the perturbations during fabrication. The resulting transmission spectrum is shown in Figure 3b. One can see the transmission peak only shifts 2 nm compared to that in Figure 2a as an indicative of the high robustness from the topological property. The electric field at the resonant peak is also highly localized in the inversion center and exponentially decay into the bulk as shown in Figure 3c. To further examine the angular robustness under fabrication perturbations, Figure 3d additionally presents the transmission spectrum versus incident angle for the structure with random thickness variations under TM polarization. The main resonant peak remains nearly fixed around 1510 nm over a broad angular range, indicating that the angle-insensitive response is largely preserved even in the perturbed case. In comparison, TE polarization does not maintain such a stable resonant wavelength with incident angle, and thus is less suitable for realizing the dispersionless bistable response studied here.

**Figure 4:**
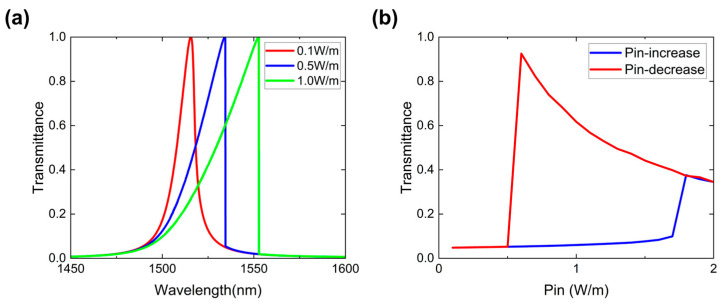
(**a**) Transmission spectra at different incident power densities. The red, blue and green curves represent the power density of 0.1 W/m, 0.5 W/m and 1.0 W/m, respectively. (**b**) Photonic hysteresis loop at the operation wavelength of 1535 nm.

Given that the Kerr nonlinear effect is strongest in the region of maximum electric field localization, we introduce the nonlinear response only into the central silicon layer at the inversion center. The relative permittivity is given as $\varepsilon_{r}=\varepsilon_{L}+\chi^{\left(3\right)}{\left|E_{loc}\right|}^{2}$, where the linear permittivity is $\varepsilon_{L}=n^{2}=12.11$, the nonlinear permittivity corresponds to $\chi^{\left(3\right)}{\left|E_{loc}\right|}^{2}$ and the Kerr coefficient is $\chi^{\left(3\right)}=6\times 10^{-11}\;m^{2}/V^{2}$ [18,19]. Thus, as the intensity of the incident light increases, the refractive indices of layers A and B increases, causing a redshift in the topological state transmission peak. As shown in Figure 4a, when the light intensity is 0.1 W/m, the transmission peak is at the wavelength of 1512 nm, and there is no influence from the nonlinearity. As the incident light intensity increases to 0.5 W/m, nonlinear effects arise, and the peak transmission shifts to 1530 nm. Continuing to increase the light intensity to 1.0 W/m causes the transmission peak to move to 1555 nm.

Based on the relationship between the bistable working wavelength and the resonant wavelength $\lambda -\lambda_{res}>\frac{\sqrt{3}}{2}FWHM$ (where $\lambda_{res}$ is the resonant wavelength, and λ is the bistable working wavelength), we scanned the optical hysteresis loop at a working wavelength of 1535 nm, within the interval of low incident light intensity (0.1 W/m) and high incident light intensity (5 W/m, where this high intensity is determined by the redshift of the resonance peak induced by nonlinearity). In Figure 4b, the red curve represents the results obtained by scanning from high to low light intensity, while the blue curve represents the results obtained by scanning from low to high light intensity. The hysteresis loop is located around 1.1 W/m, with a loop width of approximately 1.2 W/m.

## 3. Bistable PhH Device in Neural Networks

In the emerging field of photonic neural networks, the bistable function of a PhH under TM polarization conditions is used as a nonlinear activation function for optical neurons, demonstrating significant advantages in energy consumption and operating speed compared to traditional CPU and GPU neural networks. Optical nonlinear activation functions are crucial for processing complex tasks, and bistable devices, due to their high-precision pixel-by-pixel modulation capabilities and nonlinear characteristics, can effectively handle complex nonlinear patterns in image classification tasks. These devices are designed to seamlessly integrate into complex optical deep neural network architectures while being compatible with existing waveguide systems and photonic integrated circuits. Furthermore, the development of PNNs requires all-optical nonlinear activation functions, and bistable devices can easily serve as nonlinear activation functions, facilitating the integration of optical deep neural networks and advancing the further development of photonic neural networks.

To explore the application of the proposed bistable one-dimensional PhH device in all-optical neural networks, we incorporate the bistable characteristics of the PhH under TM polarization as an all-optical nonlinear activation function into the convolutional neural network simulation. To incorporate the nonlinear transmission characteristics of the device into the neural network, we construct a custom activation function based on the power transfer curve between the input and output optical signals. Specifically, the calculated discrete input/output optical power data serve as the basis, and a continuous and smooth nonlinear mapping function is obtained through cubic spline interpolation.

**Figure 5:**
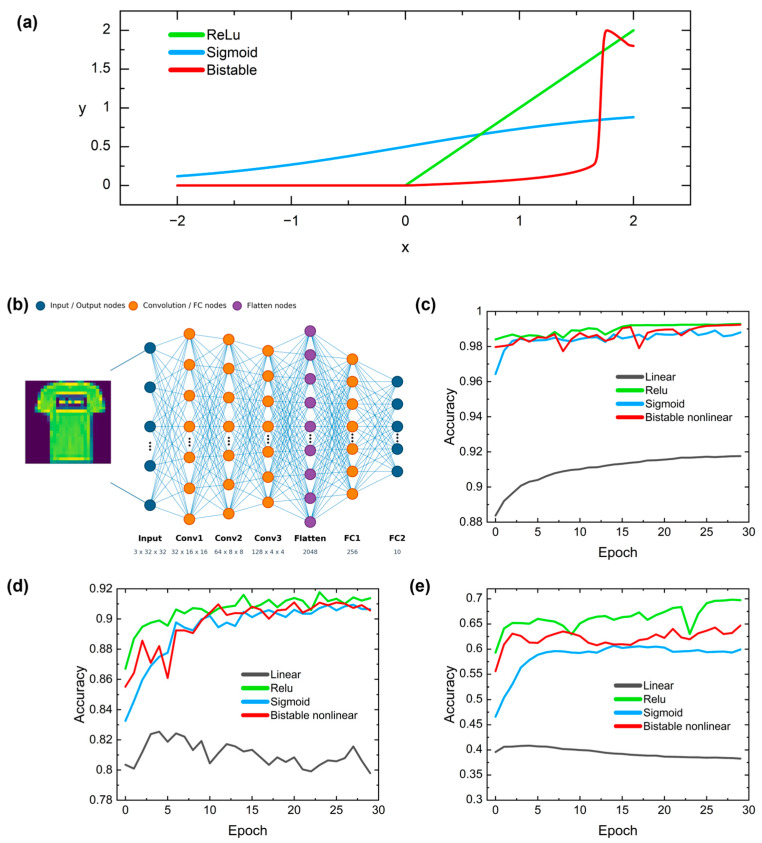
(**a**) Explicit comparison of the proposed bistable optical activation function with ReLU and Sigmoid. For the optical activation, x < 0 is mapped to y = 0, whereas x ∈ [0, 2] follows the normalized bistable device response. (**b**) A schematic of optical neural network with bistable states in PhH. (**c**–**e**) Comparison of prediction accuracy of a linear function (black), traditional nonlinear functions ReLU (green) and Sigmoid (blue), along with our proposed nonlinear function (red) based on PhH. The simulation results demonstrate that our bistable nonlinear function exhibits performance similar to that of the classic digital nonlinear activation functions, achieving 99% accuracy on the MNIST dataset, 91% on the Fashion-MNIST dataset, and 64% on the CIFAR-10 dataset. In comparison, the linear activation function produced accuracies of 91%, 81%, and 40%, respectively. The datasets are identified as follows: (**c**) MNIST, (**d**) Fashion-MNIST, and (**e**) CIFAR.

For clarity, Figure 5a explicitly compares the proposed bistable optical activation function with ReLU and Sigmoid over the same input domain. Because the physical input variable of the device is optical intensity, the original device response is defined only for nonnegative input. Accordingly, in the activation-function representation used in the neural network model, the output is set to zero for x < 0, consistent with the inactive region of ReLU. To improve training stability, an input optimization step is applied before the activation so that the effective neural network input is aligned with the calibrated operating range of the bistable optical response. This function is then embedded into the hidden layers of the neural network to describe the nonlinear modulation process of the optical signal after passing through the device. During forward propagation, the input of each hidden-layer node corresponds to the input optical intensity of the device, while the activated output corresponds to the output optical intensity. During backpropagation, gradient information is obtained by differentiating the interpolation function, thereby enabling a trainable nonlinear activation based on the physical response curve. As shown in Figure 5, our simulations utilize the MNIST, Fashion-MNIST, and CIFAR-10 datasets, conducting a comprehensive study on various tasks. The detailed model configuration is presented in the following section. The simulation results indicate that our bistable nonlinear activation function achieved performance comparable to that of traditional digital nonlinear activation functions widely used in different network architectures, such as ReLU and Sigmoid. The accuracy of the classic digital nonlinear activation functions is 99% for MNIST, 91% for Fashion-MNIST, and 68% for CIFAR-10. Our bistable nonlinear activation function reach an accuracy of 99% on MNIST, 91% on Fashion-MNIST, and 64% on CIFAR-10. In contrast, the neural network without a nonlinear activation function have recognition rates of only 92% for MNIST, 81% for Fashion-MNIST, and 40% for CIFAR-10. Notably, ReLU and Sigmoid activation functions are primarily suited to conventional neural networks implemented on CPU and GPU platforms, whereas in photonic neural networks, the activation function must be provided by the photonic device. The simulation results show that the classification accuracy achieved by our proposed nonlinear PhH is comparable to that of classical nonlinear activation functions such as ReLU and Sigmoid, indicating that it can effectively realize the nonlinear mapping capability required for neural networks. This demonstrates the feasibility and great potential of the nonlinear PhH as an activation function for photonic neural networks.

The details of the neural network model are listed as follows. In this work, a convolutional neural network classification model is implemented using PyTorch 2.1.0. The model mainly consists of three convolutional layers, one flatten layer, and two fully connected layers. The three convolutional layers have 32, 64, and 128 output channels, respectively. All convolution kernels are of size 3×3, with a stride of 2 and a padding of 1. As a result, the feature map size is progressively down sampled from 32×32 to 16×16, 8×8, and 4×4. The resulting 128×4×4 feature map is then flattened into a 2048-dimensional vector, which passes through a fully connected layer with 256 units before being mapped to a 10-dimensional output class space. During training, the Adam optimizer is used with a learning rate of 0.0003, a batch size of 64, and 30 training epochs, while the cross-entropy loss function is employed. In addition, four batch normalization (BN) layers are incorporated into the network, with three placed after the three convolutional layers and one after the first fully connected layer.

## 4. Conclusions

In this work, we propose and analyze a novel bistable device based on a dispersionless one-dimensional PhH, demonstrating extremely high nonlinearity and significant bistability. With its small footprint and fast switching speed, this device can be applied in various all-optical systems, including neural networks. The performance of the device is evaluated, and the results indicate excellent performance. Our works provide an approach towards the development of optical bistable devices characterized by dispersionless properties and enhanced stability.

## Data Availability

Data underlying the results presented in this paper are not publicly available at this time but may be obtained from the correspondence authors upon reasonable request.
